# Supplementary material for: An immunofluorescence microscopy assay to discriminate distinct expression patterns of HIV-1 Gag and Nef proteins in HIV-1 provirus-harboring cells
Source: PLoS One. 2026 Jan 16;21(1):e0340463. doi: 10.1371/journal.pone.0340463 (PMC12810823; doi:10.1371/journal.pone.0340463)
Supplement: S1 File — (PDF) [file pone.0340463.s001.pdf]

Dec 09, 2025

An immunofluorescence microscopy assay to discriminate distinct expression patterns of HIV-1 Gag and Nef protein in HIV-1 provirus-harboring cells

DOI
dx.doi.org/10.17504/protocols.io.4r3l21xqjg1y/v1

Rosana Wiscovitch-Russo¶[1], Hongyan Sui¶*[1], Mindy Smith[2], Hiromi Imamichi[2], H Clifford Lane[2], Tomozumi Imamichi[1]

[1]Laboratory of Human Retrovirology and Immunoinformatics, Applied and Developmental Research Directorate, Frederick National Laboratory for Cancer Research, Frederick, Maryland, United States of America;
[2]Laboratory of Immunoregulation, National Institute of Allergy and Infectious Diseases, National Institutes of Health, Bethesda, Maryland, United States of America

Sui-LHRI, FNL

Hongyan H Sui

OPEN ACCESS

DOI: **https://dx.doi.org/10.17504/protocols.io.4r3l21xqjg1y/v1**

Protocol Citation: Rosana Wiscovitch-Russo¶, Hongyan Sui¶*, Mindy Smith, Hiromi Imamichi, H Clifford Lane, Tomozumi Imamichi 2025. An immunofluorescence microscopy assay to discriminate distinct expression patterns of HIV-1 Gag and Nef protein in HIV-1 provirus-harboring cells. **protocols.io** **https://dx.doi.org/10.17504/protocols.io.4r3l21xqjg1y/v1**

License: This is an open access protocol distributed under the terms of the **Creative Commons Attribution License**, which permits unrestricted use, distribution, and reproduction in any medium, provided the original author and source are credited

Protocol status: Working
We use this protocol and it's working

Created: December 02, 2025

Last Modified: December 10, 2025

Protocol Integer ID: 234619

Keywords: Immunofluorescence microscopy assay, HIV-1 Gag protein, HIV-1 Nef protein , HIV-1 provirus-harboring cells, Expression pattern

Abstract

This protocol details the immunofluorescence microscopy assay to discriminate distinct expression patterns of HIV-1 Gag and Nef protein in HIV-1 provirus-harboring cells.

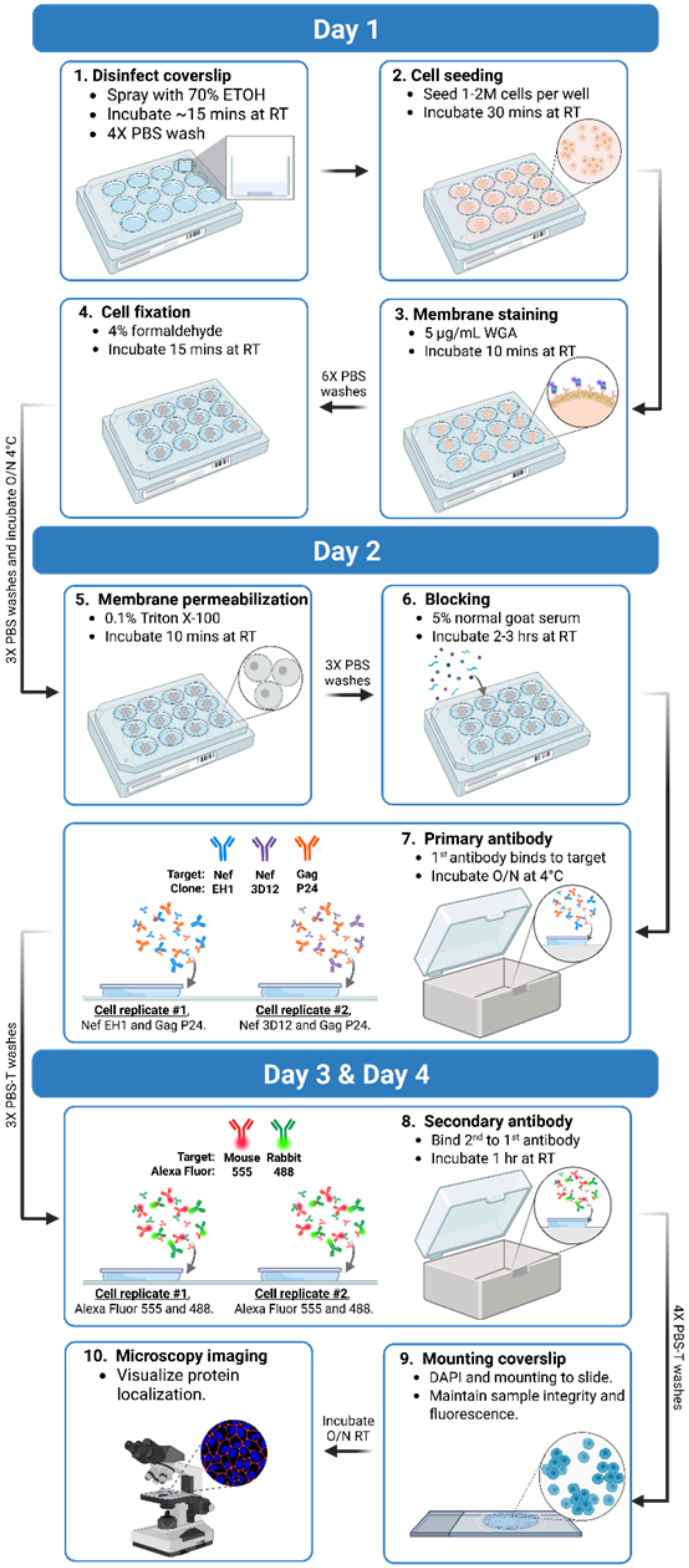

S1 Figure A. Optimized immunofluorescence workflow with WGA and DAPI staining to provide a clear boundary of [illegible]

the nuclear and cytoplasm of the cells.

Materials

Reagents

1. 70% Ethanol
2. RPMI 1640 Medium **Thermo Fisher Scientific Catalog #**11875093
3. Fetal Bovine Serum (FBS, Corning, Cat# S11150H)
4. HEPES Buffer (1 M), pH 7.3 **Quality Biological Catalog #**118-089-721
5. Gentamicin (10 mg/mL) **Thermo Fisher Catalog #**15710064
6. ViaStain AOPI Staining Solution, 5 mL **Revvity Catalog #**CS2-0106-5ML
7. Wheat Germ Agglutinin, Alexa Fluor™ 647 Conjugate **Thermo Fisher Catalog #**W32466
8. HBSS, calcium, magnesium, no phenol red **Thermo Fisher Catalog #**14025092
9. Pierce 16% Formaldehyde (w/v), Methanol-free **Thermo Fisher Scientific Catalog #**28908
10. Triton™ X-100 Surfact-Amps™ Detergent Solution **Thermo Fisher Catalog #**85111
11. 1X PBS **Quality Biological Catalog #**114-058-101
12. Tween 20, 10% aqueous solution **Merck MilliporeSigma (Sigma-Aldrich) Catalog #**11332465001
13. Intercept (PBS) Blocking Buffer **LI-COR Catalog #**927-70001
14. Mouse monoclonal anti-HIV-1 Nef clone EH1 **BEI Resources Catalog #**ARP-3689
15. Mouse monoclonal anti-HIV-1 Nef clone 3D12 **Thermo Fisher Scientific Catalog #**MA1-71501
16. Rabbit polyclonal anti-HIV-1 p24 **LifeSpan BioSciences, Inc. Catalog #**LS-C486990
17. Anti-mouse IgG (H+L), F(ab')2 Fragment (Alexa Fluor® 555 Conjugate) **Cell Signaling Technology Catalog #**4409
18. Anti-rabbit IgG (H+L), F(ab')2 Fragment (Alexa Fluor® 488 Conjugate) **Cell Signaling Technology Catalog #**4412
19. ProLong™ Diamond Antifade Mountant with DAPI **Thermo Fisher Catalog #**P36962

Consumables (General Lab Suppliers)

1. Kimwipes
2. Aluminum Foil
3. Parafilm
4. Tape
5. Clear and amber 1.5 mL microcentrifuge tubes
6. 15 mL polypropylene conical tubes
7. 10 mL barrier serological pipettes
8. 2-1000 µL barrier pipette tips
9. 12-well cell culture plates

10. 25 cm^2 flasks for culturing suspension cells with filter cap
11.

BioCoat® Poly-L-Lysine 12 mm #1 German Glass Coverslip, 40/Pack, 80/Case **Corning Catalog #**354085

12. Microscope slide
13. Cellometer SD100 Slides, box of 75 slides **Revvity Catalog #**CHT4-SD100-002
14. Silica gel beads

Equipment (General Lab Suppliers)

1. Excelta DN-3C-SA-SE tweezers with fine hook, which is created by bending one tip toward outside, to lift fragile glass coverslips
2. Large plastic box (approximately 15.2 × 10.2 × 3.2 cm)
3. 37°C cell incubator with 5% CO_2
4. 37°C water bath
5. Vortex
6. Bench top centrifuge
7. 4°C lab refrigerator
8. -20°C lab freezer
9. 2-1000 µL single channel pipettes
10. Serological pipette controller
11. Cellometer Automated Cell Counter (Revvity, Waltham, Massachusetts, United States)
12. Light microscope
13. Confocal microscope

Troubleshooting

Prepare Solutions

15m

1 **Complete Growth Medium:**

- To 500 mL of RPMI-1640 (Cat# 11875-093) add 50 mL of FBS (final concentration 10%), 12.5 mL of HEPES buffer (final concentration [M] 25 millimolar (mM)), and 500 µL of gentamicin (final concentration [M] 10 µg/mL).
- Before use, warm the complete growth medium in a 37 °C water bath for approximately 00:30:00 .

2 **WGA Alexa Fluor 647 Stock Solution ([M] 1.0 mg/mL):**

10m

- In the company provided tube, dissolve 5 mg of lyophilized WGA in 5 mL of 1x PBS at Room temperature for 00:10:00 .
- Mix and aliquot 250 µL into 1.5 mL amber tubes. Store the aliquots at -20 °C for long-term storage, avoid freeze-thaw cycles.

Note

WGA Alexa Fluor 647 is light-sensitive and must be protected from light.

3 **WGA Alexa Fluor 647 Working Solution (5.0 µg/mL):**

5m

- Begin by vortexing and centrifuging the WGA stock solution ([M] 1.0 mg/mL) at 300 x g for 00:03:00 - 00:05:00 .
- In a light-protected tube, dilute the stock solution 1:200 in HBSS (e.g. add 5 µL of stock in 1 mL HBSS). Vortex thoroughly and briefly centrifuge the solution before use.

Note

Avoid pipetting from the bottom of the tube, as the WGA solution may contain undissolved reagent that can cause high fluorescence background during downstream sample analysis.

4 **Fixation Solution:**

- Prepare 4% formaldehyde solution in a 15 mL conical tube.

- Before use dilute 16% formaldehyde solution 1:4 in HBSS (e.g. 1 mL of formaldehyde into 3 mL of HBSS), then vortex to mix thoroughly.

Note

Formaldehyde is light-sensitive and must be prepared fresh before use and protected from light.

5 **Permeability Solution:**

- Prepare 0.1% Triton X-100 solution in a 15 mL conical tube.
- Before use dilute 10% Triton X-100 solution 1:100 in 1x PBS (e.g. add 10 µL of Triton X-100 into 1 mL of 1x PBS), then vortex to mix thoroughly.
- Prepare fresh on the day of use to maintain detergent efficacy.

6 **Wash Buffer:**

- Prepare 0.1% PBS-Tween (PBS-T) as follows: add 5 mL of 10% of Tween to 500 mL of 1x PBS.
- Shake to mix and store at Room temperature .

Protocol - Seeding cells onto glass coverslip (day 1)

40m

7 Grow H9 cells and H9MN, H9MN-FI and H9MN-FD in 25 cm^2 tissue culture flask with complete growth medium until the cells reached confluency. Refer to manufacture's detailed product information for handling and subculturing H9 or HIV-1 provirus harboring H9 cells.

8

- Using a tweezer, lift the poly-l-lysine-coated coverslip from its storage case and disinfect it by spraying both sides with 70% ethanol.
- Remove excess ethanol by gently touching the edge of the coverslip to a clean Kimwipe. Then, place the coverslip into a 12-well plate, leaning it against the wall to prevent it from sticking to the bottom of the well.
- Incubating at Room temperature for approximately 10-15 minutes until the ethanol has evaporated.
- See **S1 Figure B** and refer to **Step 8** for an example of the coverslip position in the well.

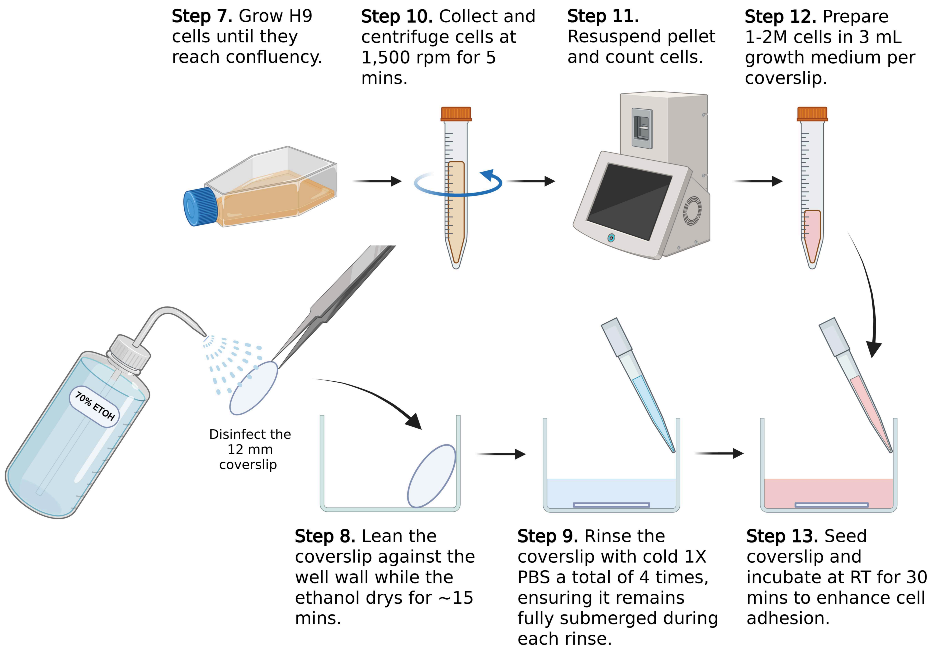

S1 Figure B. Workflow showing how to disinfect and seed cells onto the poly-l-lysine-coated coverslip.

Note

since both Anti-Nef originate from the same mouse source, each H9 cell line must be seeded on two separate coverslips and treated independently for the respective Nef antibody. Consequently, ensure an adequate number of coverslips are prepared. In other words, one sample needs prepare two coverslips.

9 Remove remaining ethanol from the coverslip by rinsing with 1 mL - 2 mL of cold 1x PBS and incubate at Room temperature for 00:05:00 . Remove the buffer and repeat wash for a total of four times. 5m

Note

ensuring the coverslip remains fully submerged during each rinse. Ready to use, set aside the disinfected coverslips in 1-2 mL of 1x PBS.

10 Transfer cells from flask into a 15 mL conical tube and pellet by centrifuging at 1.500 rpm, Room temperature, 00:05:00 . 5m

11 Remove and discard cell supernatant. Then resuspend the pellet in 3 mL of complete growth medium and proceed to cell counting using ViaStain AOPI staining solution, Cellometer slides and automated cell counter according to manufacturer's

instructions (https://www.revvity.com/product/vs-cellometer-aopi-staining-solution-cs2-0106-5ml).

12 For each coverslip, prepare approximately 1-2 million cells in 3 mL of complete growth medium.

Note

include additional volume to account for pipetting error while preparing the cells for multiple wells.

13 Remove the buffer from the prepared coverslips (see step 9). Add 3 mL of the diluted cells suspension to the designated coverslip wells and incubate at Room temperature for 00:30:00 to enhance adhesion of the cells onto the lysine-coated coverslip. 30m

Note

during the incubation period, prepare WGA working solution (5.0 µg/mL) and fixation solution (refer to Prepare Solutions section).

14 After 00:30:00 , the cells should adhere to the lysine-coated coverslip. Proceed to WGA staining.

Protocol - WGA membrane staining before cell fixation (day 1) 35m

15 Without disturbing the adhered cells, carefully remove the supernatant by aspirating from the bottom of the well, away from the coverslip.

16 Without disturbing the adhered cells, gently add 1 mL of WGA working solution (5.0 µg/mL) by slowly dispensing along the top inner wall of the well (see **S1 Figure B** and refer to **Step 9** for an example of the pipette tip location). Protect the coverslip from light by covering the 12-well plate with aluminum foil and incubate at Room temperature for 00:10:00 . 10m

17 Carefully remove the solution and gently rinse the coverslip with 2 mL of 1x PBS by slowly dispensing along the top inner wall of the well. Incubate at Room temperature for 00:05:00 , protected from light. Repeat the wash a total of six times to remove excess or unbound WGA. 5m

18 Remove the buffer and gently add 1 mL of fixing solution to each well by slowly dispensing along the top inner wall of the well. Incubate at Room temperature for 00:15:00 , protected from light. 15m

19 Carefully remove the solution and gently rinse the coverslip with 2 mL of 1x PBS. Incubate at Room temperature for 00:05:00 , protected from light. Repeat the wash a total of three times. 5m

20 Store the coverslips Overnight in 1x PBS at 4 °C .

Note

This is an optional stopping point. If time permits, please proceed directly to Step 21.

Protocol - Permeabilization, blocking and primary antibody staining (day 2) 15m

21 Using tweezers and a 200 µL pipette tip, carefully transfer the coverslips into a new 12-well plate containing 1 mL of 1x PBS in each well. Place each coverslip **cell side up** into the designated wells with 1x PBS. Refer to **S1 Figure C** for guidance on lifting and transferring the fragile glass coverslips from a 12-well plate.

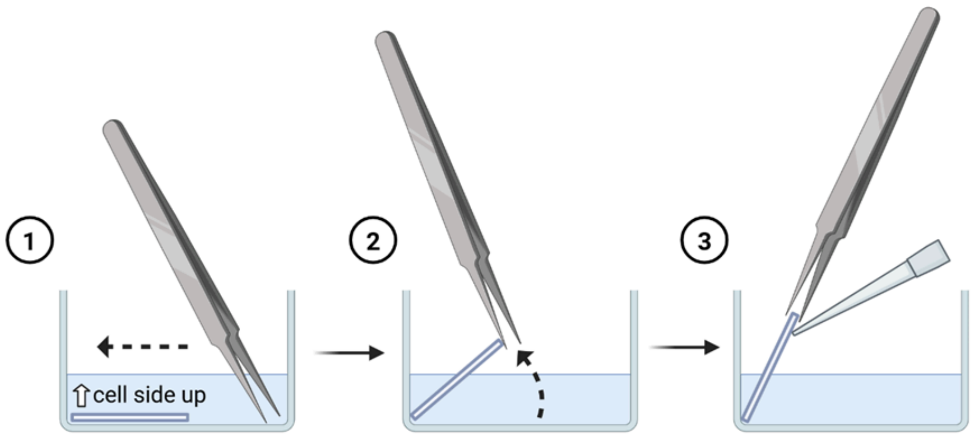

S1 Figure C. The illustration shows how to avoid scratching or breaking the fragile glass coverslip while transferring the coverslips from a 12-well cell culture plate.(1) Slowly drag the tweezers from the top edge of the well toward the center, carefully feeling for the edge of the coverslip. **(2)** Using the hooked tip of the tweezers, gently lift the coverslip. **(3)** Meanwhile, use a pipette tip to hold the coverslip in place while securing its edge with the tweezers.

22 Once all the coverslips have been transferred into a new 12-well plate, remove the buffer and gently add 1 mL of permeability solution to each well. Incubate at Room temperature for 00:10:00 protected from light.

10m

Note

Prepare a fresh permeability solution following the instructions provided in the Prepare Solutions section and use the solution immediately after preparation for best results.

23 Remove the solution and rinse the coverslip with 2 mL of 1x PBS. Incubate at Room temperature for 00:05:00 on a rocker with gentle agitation, protected from light. Repeat the wash a total of three times.

5m

24 Remove the buffer and add 2 mL of Intercept (PBS) Blocking Buffer. Incubate at Room temperature for at least 01:00:00 on a rocker with gentle agitation, protected from light.

25 During the incubation period, in a clear 1.5 mL microcentrifuge tube, prepare 100 µL of the primary antibody in Intercept (PBS) Blocking Buffer per coverslip. Dilute the antibody according to manufacturer's instructions (see **S1 Table A**).

	A	B	C	D	E	F
	**Primary Antibody**	**Clone**	**Company**	**Cat#**	**Source**	**Dilution**
	Anti-Nef	EH1	AIDS Reagent Program	3689	Mouse	1:100
	Anti-Nef	3D12	Thermo Fisher Scientific	MA1-71501	Mouse	1:50
	Anti-HIV-1 P24	polyclonal	LS Bio	LS-c486990	Rabbit	1:200

S1 Table A. A list of primary antibodies and their dilutions in the assay.

Note

since both Anti-Nef originate from the same mouse source, each type of cells must be seeded on two separate coverslips and treated independently. For instance, cell replicate #1 with anti-Nef EH1 and anti-P24 antibodies, and cell replicate #2 with anti-Nef 3D12 and anti-P24 antibody (**S1 Figure A**). Include extra volume of diluted antibody while staining multiple coverslips. Refer to **S1 Figure A** for guidance on primary antibody staining strategy.

26 Prepare a large plastic box (e.g. the box used for western blot incubation) by lining the bottom with Parafilm and securing it in place with tape. Ensure there is adequate spacing between regions and label each coverslip region accordingly.

27 On the secured Parafilm, dispense an 80 µL droplet of the prepared primary antibody into the designated region.

28 Using tweezers and a 200 µL pipette tip, carefully transfer the coverslips **cell side down** onto the primary antibody droplet.

- Before placement, gently touch the edge of the coverslip to a clean Kimwipe to remove excess buffer. Then, slowly lower the coverslip over the antibody droplet to avoid introducing air bubbles. Refer to **S1 Figure C** for guidance on lifting and transferring the fragile glass coverslips from a 12-well plate.

29 Incubate the coverslips with the primary antibody Overnight at 4 °C, protected from light.

Note

to maintain a humid environment and prevent the coverslips from drying out, place a damp DI water-soaked Kimwipe in the corner of the western blot box housing the coverslips.

Protocol - 2nd antibody staining (day 3)

10m

30 The following morning, using tweezers and a 200 µL pipette tip, carefully transfer the coverslips into a new 12-well plate containing 2 mL of wash buffer (PBS-T). Place each coverslip **cell side up** into the designated wells. Refer to **S1 Figure D** for guidance on lifting and transferring the fragile glass coverslips from the parafilm lined box.

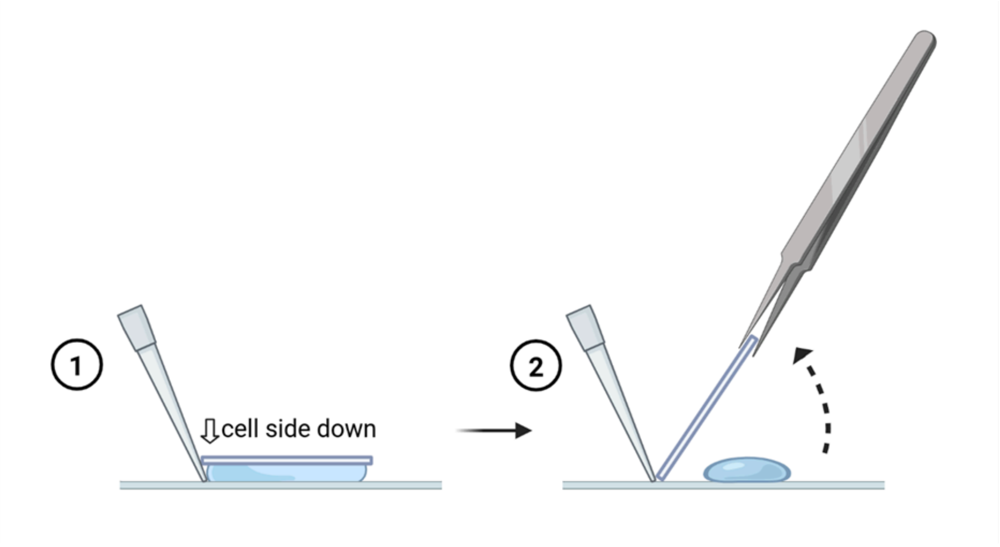

S1 Figure D. The illustration shows how to transfer the coverslip from the primary antibody staining box to the 12-well cell culture plate for washing. To avoid dragging and potentially removing cells from the coverslip. (1) Place the pipette tip at the back edge of the coverslip to hold it in place. (2) Then, gently securing and lifting its edge with the tweezers.

31 Once all the coverslips have been transferred into the new 12-well plate with 1 mL - 2 mL PBS-T filled in, incubate at Room temperature for 00:05:00 on a rocker with gentle agitation, protected from light. Repeat the PBS-T wash a total of three times to remove unbound primary antibody. 5m

32 During the incubation period, in an amber 1.5 mL microcentrifuge tube, prepare 100 µL of the secondary antibody (listed in **S1 Table B**) in Intercept (PBS) Blocking Buffer per coverslip. Dilute the antibody according to manufacturer's instructions:

	A	B	C	D	E
	**Target**	**Secondary Antibody**	**Company**	**Cat#**	**Dilution**
	Mouse	Alexa Fluor 555 goat anti-mouse IgG	Cell Signaling	4409S	1:1000
	Rabbit	Alexa Fluor 488 goat anti-rabbit IgG	Cell Signaling	4412S	1:1000

S1 Table B. A list of secondary antibodies and their dilutions in the assay.

33 Prepare a large plastic box by lining the bottom with Parafilm and securing it in place with tape. Ensure there is adequate spacing between regions and label each coverslip region

accordingly.

34 On the secured Parafilm, dispense an 80 µL droplet of the prepared secondary antibody into the designated region.

35 Using tweezers and a 200 µL pipette tip, carefully transfer the coverslips **cell side down** onto the secondary antibody droplet. Before placement, gently touch the edge of the coverslip to a clean Kimwipe to remove excess buffer. Then, slowly lower the coverslip over the antibody droplet to avoid introducing air bubbles. Refer to **S1 Figure C** for guidance on lifting and transferring the fragile glass coverslips from a 12-well plate.

36 Incubate the coverslips with the secondary antibody at Room temperature for an hour, protected from light.

37 Using tweezers and a 200 µL pipette tip, carefully transfer the coverslips into a new 12-well plate containing 2 mL of PBS-T. Place each coverslip **cell side up** into the designated wells. Refer to **S1 Figure D** for guidance on lifting and transferring the fragile glass coverslips from the parafilm lined box.

38 Once all the coverslips have been transferred into the new 12-well plate, incubate at Room temperature for 00:05:00 on a rocker with gentle agitation, protected from light. Repeat the PBS-T wash a total of four times to remove unbound secondary antibody. 5m

39 After the cells have been treated for immunostaining, proceed to mount the coverslips onto microscope slides.

Protocol - Mounting coverslip (Day 3 & Day 4)

40 Clean and remove dust particles from the microscope slides by spraying them with 70% ethanol and wiping off the excess with a Kimwipe. Then, label each slide with the date, antibodies, and the sample names in order.

41 Prepare a large plastic box by lining the bottom with absorbent paper and securing it in place with tape. Place a smaller clear container filled with silica gel beads along the edge of the box to absorb excess moisture from the environment.

42 Add one drop of ProLong™ Diamond Antifade Mountant with DAPI onto the clean microscope slide for each coverslip. Avoid introducing bubbles; if bubbles are present, gently remove them using a pipette.

43 Using tweezers and a 200 µL pipette tip, carefully transfer the coverslips **cell side down** onto the DAPI droplet. Before placement, gently touch the edge of the coverslip to a clean Kimwipe to remove excess buffer. Then, slowly lower the coverslip over the DAPI

droplet to avoid introducing air bubbles. Refer to **S1 Figure C** for guidance on lifting and transferring the fragile glass coverslips from a 12-well plate.

44 Protect the coverslip from light by covering the box with aluminum foil and placing into a dark cabinet. Incubate the mounted coverslips Overnight at Room temperature .

45 The following morning of day 4, the mounted coverslips are ready for confocal microscopic observation.
