# Supplementary material for: An immunofluorescence microscopy assay to discriminate distinct expression patterns of HIV-1 Gag and Nef proteins in HIV-1 provirus-harboring cells
Source: PLoS One. 2026 Jan 16;21(1):e0340463. doi: 10.1371/journal.pone.0340463 (PMC12810823; doi:10.1371/journal.pone.0340463)
Supplement: S1 Table — (PDF) [file pone.0340463.s002.pdf]

S1 Table. A list of primary antibodies screened in the study.

No.	Target	Host	Clone	Company	Catalog#	Isotype	Dilution
**N1**	Nef	Mouse	JR6	Abcam	ab42358	lgG1	1:500
**N2**	Nef	Mouse	3A2	Thermo Fisher	MA1-71504	lgG1	1:50
**N3**	Nef	Mouse	3D12	Thermo Fisher	MA1-71501	lgG1	1:50
**N4**	Nef	Rabbit	polyclonal	Abcam	ab63918	lgG	1:100
**N5**	Nef	Rabbit	polyclonal	NIH HIV Reagent Program	Not available		1:50
**N6**	Nef	Mouse	ABM069	Thermo Fisher	603-610	lgG2a	1:200
**N7**	Nef	Mouse	ABM068	Thermo Fisher	603-390	lgG3	1:200
**N8**	Nef	Mouse	ABM067	Thermo Fisher	603-400	lgG1	1:200
**N9**	Nef	Mouse	3E6	Thermo Fisher	MA1-71503	lgG1	1:100
**N10**	Nef	Mouse	2H12	Thermo Fisher	MA1-71505	lgG1	1:100
**N11**	Nef	Mouse	N5	Thermo Fisher	MA1-71507	lgG1	1:50
**N12**	Nef	Mouse	EH1	NIH HIV Reagent Program	3689	IgG1	1:100
**G1**	Gag	Rabbit	EPR27327-41	Abcam	ab308070	lgG	1:2000
**G2**	P24	Rabbit	002	Thermo Fisher	MA5-29967	lgG	1:100
**G3**	Gag	Rabbit	Polyclonal	LS Bio	LS-C486990	lgG	1:50
**G4**	Gag/ P24	Rabbit	S1298DS	Creative Biolabs	MOR-4219	lgG	1:100
