# Supplementary material for: An immunofluorescence microscopy assay to discriminate distinct expression patterns of HIV-1 Gag and Nef proteins in HIV-1 provirus-harboring cells
Source: PLoS One. 2026 Jan 16;21(1):e0340463. doi: 10.1371/journal.pone.0340463 (PMC12810823; doi:10.1371/journal.pone.0340463)
Supplement: Attachment — Submitted filename: Response to reviewers.docx [file pone.0340463.s005.docx]

**Response to the editor and reviewers**

**Comments from Editor**

*Journal Requirements:*

*When submitting your revision, we need you to address these additional requirements.*

*1.Please ensure that your manuscript meets PLOS ONE's style requirements, including those for file naming. The PLOS ONE style templates can be found at*

[*https://journals.plos.org/plosone/s/file?id=wjVg/PLOSOne_formatting_sample_main_body.pdf*](https://journals.plos.org/plosone/s/file?id=wjVg/PLOSOne_formatting_sample_main_body.pdf) *and*

[*https://journals.plos.org/plosone/s/file?id=ba62/PLOSOne_formatting_sample_title_authors_affiliations.pdf*](https://gcc02.safelinks.protection.outlook.com/?url=https%3A%2F%2Ftrack.editorialmanager.com%2FCL0%2Fhttps%3A%252F%252Fjournals.plos.org%252Fplosone%252Fs%252Ffile%253Fid%3Dba62%252FPLOSOne_formatting_sample_title_authors_affiliations.pdf%2F1%2F010f019a6c58bfca-c38974d4-e4ec-4722-823d-7bfc77ddf38a-000000%2FyQ0t3I3_Z6iC0_A-IQ5jTq04wu9zMJuy4ani54trVS0%3D236&data=05%7C02%7Chongyan.sui%40nih.gov%7Cffe8412c40934a1f4e1608de201e4435%7C14b77578977342d58507251ca2dc2b06%7C0%7C0%7C638983511834955617%7CUnknown%7CTWFpbGZsb3d8eyJFbXB0eU1hcGkiOnRydWUsIlYiOiIwLjAuMDAwMCIsIlAiOiJXaW4zMiIsIkFOIjoiTWFpbCIsIldUIjoyfQ%3D%3D%7C0%7C%7C%7C&sdata=L6%2Fsb4kDvzhFfvls8TR9kTAjwE%2BuRXm5NuxopW91gfo%3D&reserved=0)

- ***Response:*** *The following changes were made to the manuscript to meet PLOS ONE’s style requirements:*

a) Title page affiliations: Department, Institution, City, State, Country.
b) Title page: corresponding author’s initials in parentheses after email address.
c) Changed font size for Level 1 and 2 Heading to 18 and 16 pt font.
d) Moved the figure captions after the paragraph in which they are first cited.
e) The Figs 1-3 were saved in individual tiff files.
f) The supporting information was moved after the references.

*2. We note that the grant information you provided in the ‘Funding Information’ and ‘Financial Disclosure’ sections do not match.*

*When you submit, please ensure that you provide the correct grant numbers for the awards you received for your study in the ‘Funding Information’ section.*

- **Response:** The grant information has provided in the “Funding information” section while resubmission.

*3. Thank you for stating the following financial disclosure:*

*“**This project has been funded in whole or in part with federal funds from the National Cancer Institute, National Institutes of Health, under contract number HHSN261200800001E.”*

*Please state what role the funders took in the study.  If the funders had no role, please state: "The funders had no role in study design, data collection and analysis, decision to publish, or preparation of the manuscript."*

*If this statement is not correct you must amend it as needed.*

*Please include this amended Role of Funder statement in your cover letter; we will change the online submission form on your behalf.*

- **Response:** The role of funder statement will provide together with the funding information. All that information is included in the cover letter.

Here is the amended statement “This project has been funded in whole or in part with federal funds from the National Cancer Institute, National Institutes of Health (NIH), under contract number HHSN261200800001E. The founders had no role in study design, data collection and analysis, decision to publish, or preparation of the manuscript. The contributions of the Mindy Smith, Hiromi Imamichi, and H. Clifford Lane are considered Works of the United States Government. The findings and conclusions presented in this paper are those of the authors and do not necessarily reflect the views of the NIH or the U.S. Department of Health and Human Services”.

*4. Thank you for stating the following in the Acknowledgments Section of your manuscript:*

*“The following reagent was obtained through the NIH HIV Reagent Program, Division of 189 AIDS, NIAID, NIH: Human Immunodeficiency Virus 1 (HIV-1) MN-Infected H9 Cells, ARP-402, 190 contributed by Dr. Robert Gallo; Monoclonal Anti-Human Immunodeficiency Virus Type 1 (HIV191 1) Nef Protein (EH1), ARP-3689, contributed by Dr. James Hoxie. All illustrations and workflows 192 were created using BioRender.com. This project has been funded in whole or in part with 193 federal funds from the National Cancer Institute, National Institutes of Health, under contract 194 number HHSN261200800001E. This research was supported in part by the Intramural Research 195 Program of the National Institutes of Health (NIH). The contributions of the Mindy Smith, Hiromi 196 Imamichi, and H. Clifford Lane are considered Works of the United States Government. The 197 findings and conclusions presented in this paper are those of the authors and do not necessarily 198 reflect the views of the NIH or the U.S. Department of Health and Human Services.”*

*We note that you have provided funding information that is not currently declared in your Funding Statement. However, funding information should not appear in the Acknowledgments section or other areas of your manuscript. We will only publish funding information present in the Funding Statement section of the online submission form.*

*Please remove any funding-related text from the manuscript and let us know how you would like to update your Funding Statement. Currently, your Funding Statement reads as follows:*

*“This project has been funded in whole or in part with federal funds from the National Cancer Institute, National Institutes of Health, under contract number HHSN261200800001E.”*

*Please include your amended statements within your cover letter; we will change the online submission form on your behalf.*

- **Response:** The Funding statement “This project has been funded in whole or in part with federal funds from the National Cancer Institute, National Institutes of Health, under contract number HHSN261200800001E.” is correct. All that information has been removed from acknowledgement section of the manuscript.

*5. We note you have not yet provided a protocols.io PDF version of your protocol and/or a protocols.io DOI. When you submit your revision, please provide a PDF version of your protocol as generated by protocols.io (the file will have the protocols.io logo in the upper right corner of the first page) as a Supporting Information file. The filename should be S1_file.pdf, and you should enter “S1 File” into the Description field. Any additional protocols should be numbered S2, S3, and so on. Please also follow the instructions for Supporting Information captions [https://journals.plos.org/plosone/s/supporting-information#loc-captions]. The title in the caption should read: “**Step-by-step protocol, also available on protocols.io.”*

*Please assign your protocol a protocols.io DOI, if you have not already done so, and include the following line in the Materials and Methods section of your manuscript: “The protocol described in this peer-reviewed article is published on protocols.io (*[*https://dx.doi.org/10.17504/protocols.io*](https://dx.doi.org/10.17504/protocols.io)*.[...]) and is included for printing purposes as S1 File.” You should also supply the DOI in the Protocols.io DOI field of the submission form when you submit your revision.*

*If you have not yet uploaded your protocol to protocols.io, you are invited to use the platform’s protocol entry service [https://www.protocols.io/we-enter-protocols] for doing so, at no charge. Through this service, the team at protocols.io will enter your protocol for you and format it in a way that takes advantage of the platform’s features. When submitting your protocol to the protocol entry service please include the customer code PLOS2022 in the Note field and indicate that your protocol is associated with a PLOS ONE Lab Protocol Submission. You should also include the title and manuscript number of your PLOS ONE submission.*

- **Response:** the step-by-step protocol has been uploaded to protocols.io. here is the link: <https://dx.doi.org/10.17504/protocols.io.4r3l21xqjg1y/v1>.

*6. If the reviewer comments include a recommendation to cite specific previously published works, please review and evaluate these publications to determine whether they are relevant and should be cited. There is no requirement to cite these works unless the editor has indicated otherwise.* 

- **Response:** Reviewer 2 cited one publication as an example and was not included in this manuscript.

*Additional Editor Comments:

Your manuscript was reviewed by two experts and there is agreement your protocol has some merit. However, revisions explicitly as stated by reviewer 1 are necessary. Especially, you should present the manuscript strictly in a style that is adequate for a protocol.*

- **Response:** Additional analysis or revisions have been incorporated into the manuscript according to reviewer 1’s comments. Please refer to the response to reviewer 1 section for the details.

**Reviewer comments: Reviewer 1**

*Reviewer #1: Wiscovitch-Russo et al. set out to develop an immunofluorescence assay that identifies Nef and Gag in cells containing HIV proviruses. After determining the optimal order for WGA staining – prior to fixation – for identification of the plasma membrane, they screened fourteen anti-Nef antibodies and four anti-HIV-1 p24 antibodies. The anti-Nef clones EH1 and 3D12 were selected and subsequently utilized in cells containing intact proviruses (H9MN-FI), a mixture of intact and defective proviruses (H9MN), and only a single defective proviral clone (H9MN-FD). H9 cells containing no proviruses served as a negative control. Consistently, the antibody EH1 clone was associated with cytoplasmic Nef expression, while the 3D12 clone signal was membrane localized. Notably, Nef in H9MN-FD cells did not localize at the plasma membrane, indicating that proteins translated from defective proviruses may be altered from their intact-proviral derived counterparts.
The following revisions would improve the manuscript.
1. The authors have presented a manuscript as a “Protocol”, yet most of the methods are in supplemental materials. This information should be in the main text for this type of format.*

- **Response:** We appreciate the reviewer’s insight regarding the placement of methodological details. PLOS ONE’s “Lab Protocol” format requires that the full step-by-step protocol be provided in a supplemental file (S1 File) and posted on Protocols.io, while the main manuscript focuses on the overview, rational, validation and discussion. In accordance with these guidelines, we have included the detailed protocol in the supplemental file and provided the Protocols.io link (<https://dx.doi.org/10.17504/protocols.io.4r3l21xqjg1y/v1> ) in the revised manuscript.

*2. The authors should more clearly explain why they tested EH1 and 3D12 anti-Nef antibody clones in the intact provirus H9s (H9MN-FI).*

- **Response:** The H9MN-FI cell line contains a single clone harboring an intact HIV provirus and therefore serves as an appropriate positive control for establishing and optimizing our immunofluorescence assay. In this control, both EH1 and 3D12 antibodies yield positive signals, allowing us to screen and refine detection conditions. To prevent any confusion regarding the use of this control, we have added additional description about the H9MN-FI cells in the Results section as shown in page 7 and lines 123-125 in the revised manuscript.

*3. Pearson Correlation plots: These correlation plots are from analysis of individual cells/images – since the authors do not disclose how many cells they analyzed per condition, I’m assuming they analyzed the cells within the region of interest depicted in their images (Fig 2 C & D) – could benefit from clarification
o Again for the Pearson Correlation plots, a bigger sample size should be included; averaging out the PCC value will give more validity to their results.*

- **Response:** We appreciate the reviewer’s insightful suggestions regarding the colocalization analysis. In response, we have re-performed the analysis using a “none” ROI definition, which allowed us to include a larger sample size. The updated analysis was conducted using at least three independent confocal images. Colocalization coefficients are now presented as mean ± SD, and statistical significance was assessed using a t-test. The resulting p-value (p = 0.0151) is below 0.05, indicating a statistically significant difference. Please refer to the specified page 7 and lines 133-138 in the revised manuscript for detailed updates.

*• For Figure 3, the authors should also perform the colocalization analysis with the H9MN cells with the mixed intact and defective proviral clone populations, and the H9MN-FD cells with only defective proviruses – to bolster their observations that there are differences in Nef localization*

- **Response:** We thank the reviewer for this suggestion. The colocalization analysis was performed only in H9MN-FI cells, which harbor a single intact HIV provirus and serve as a positive control. In these cells, both EH1 and 3D12 antibodies detect Nef, and only the Nef signal from 3D12 colocalizes with Gag, allowing us to evaluate antibody-specific Nef localization.

In H9MN and H9MN-FD cells, which contain mixed or exclusively defective proviruses, Gag localization is also disrupted compared with H9MN-FI. Therefore, colocalization analysis of these cells would not provide reliable or interpretable information regarding Nef localization. Our goal is to use established positive control to guide interpretation of Nef detection patterns in the test samples rather than to rely on colocalization as a direct readout.

*4. For immunostaining protocols, the authors should include antibody dilution info.*

- **Response:** The antibody dilution information has been made available in the supporting information file S1 Table A (Primary antibodies), S1 Table B (2^nd^ antibodies), and S2 Table (all screened antibodies).

*5. For the Imaging and Analysis:
• What plug-in within Fiji was used to do the colocalization analysis? (either Coloc 2 or JACoP)?*

- **Response:** We performed colocalization analysis using the colocalization threshold plug-in within Fiji.

*• The authors should define what metrics they are using to quantify colocalization (i.e. PCC, MOC)*

- **Response:** Within colocalization threshold, we quantified colocalization using the Rcoloc metric, which represents the Pearson’s correlation coefficient (PCC) calculated only within the thresholded colocalized region identified by the algorithm. This value reflects the linear correlation between the two fluorescence channels specifically in voxels where both signals are present. In addition, colocalization threshold also reports the global PCC, and the Mander’s overlap coefficients (M1 and M2) can also be calculated if needed; however, our analysis relied on Rcoloc, as it more stringently reflects the correlation within the overlapping signal volumes.

*• Need to include the software versions they utilized – for both ZEN and Fiji*

- **Response:** Images were initially processed using ZEN software 3.1 (blue edition).

All above information has been added to the Materials and method section, please refer to page 5 and lines 85-96 for the details.

*6. For the quantification via Nef and WGA stain colocalization analysis:
Could the lack of membrane localization in the H9MN-FD condition have to do with some alteration to the N-terminal myristoylation*

- **Response:** In response to the reviewer’s comment, we added the following statement and references to the manuscript:

“Notably, the anti-HIV-1 Nef clone 3D12 targets conserved epitope in the Nef N-terminal close to the myristoyl group and myristoylation of Nef protein is essential for membrane association [25, 26]. For the defective provirus in H9MN-FD, it is plausible that the 1-bp insertion in the RT gene may or may not be directly involved in the observed changes in Nef protein myrisoylation and localization”.

Please refer to page 9 and lines 174-179 in revised manuscript for the details.

*Minor Comments:
Line 40: This latent reservoir is a major barrier to a cure*

*Line 43: ‘Intact’ should not be capitalized*

*Line 48/49: Replace ‘HIV-infected individuals’ with ‘people living with HIV-1’*

*Line 52: No need to define Group-specific antigen and Negative regulatory factor –*

*Line 54: Switch order of ‘accessory multifunctional’ → multifunctional accessory protein*

*Line 56: Already mentioned that Nef is an accessory protein in the previous sentence; would remove ‘is an auxiliary protein’*

*Line 76: Verb tense change – should be ‘reached’ instead of reach*

*Line 145: ‘It’ should be lowercase*

- **Response:** All minor revisions mentioned above have been incorporated into the manuscript.

**Reviewer comments: Reviewer 2**

*Overview: This is a timely and interesting methods paper given the emerging picture of the clinical relevance of defective proviruses in the HIV-1 latent reservoir. The paper describes a staining protocol using two antibodies to HIV-1 Nef that can differentiate proteins produced from intact and defective HIV-1 proviruses in combination with an anti-HIV-1 Gag antibody. The manuscript is concise but clear and was a pleasure to read. The protocol is clear and easy to understand with a good level of detail for replicating the experiments in other settings. Most of the points in the revision document are recommend small changes (like typos) to the manuscript and supplementary information. However, point 10 is important to note for translation of this protocol into one that can be used with clinical samples.*

1. *Line 39 (optional): Suggestion to change the word “targeted” to “removed” or a synonym. There is some (ex vivo) evidence of CTL recognition of cells with defective genomes if proteins are expressed, even if that targeting does not effectively clear the reservoir in people with HIV (DOI:* [*10.1016/j.chom.2017.03.008*](https://doi.org/10.1016/j.chom.2017.03.008) *).*

- **Response:** Thanks for the comment. The suggested change has been made in the	manuscript.

2. Line 67: Please add the catalogue number for H9NM cells (ARP-402).

- **Response:** The H9MN cells catalogue number has been incorporated into the materials and methods section under cells and reagents in page 4, line 69 of revised manuscript.

3. Line 76: Please change “reach to “reached”.

- **Response:** The suggested change has been made in the manuscript.

4. Line 82: Kindly remember to include the Protocols.io link in the final version.

- **Response:** The link of proteocl.io (<https://dx.doi.org/10.17504/protocols.io.4r3l21xqjg1y/v1> ) has been included in the final version of the manuscript.

5. *Can the authors please add a caveat to the conclusion about cross-subtype recognition of the Gag and Nef antibodies used in this experiment? This speaks directly to the how broadly this assay can be used to characterize cells harbouring intact and defective proviruses across HIV subtypes which are distributed geographically.*

- **Response:** Thank you for the insightful suggestions. We have added the following statement to the manuscript:

“Nef’s N- and C-terminal regions are partially conserved but can vary among HIV-1 subtypes and isolates. Because EH1 and 3D12 recognize short linear or terminal epitopes, even single amino acid changes in these regions may disrupt antibody binding. This assay was developed using cell lines carrying one of the most widely studied and prevalent HIV-1 variants (Group M, Subtype B). Nevertheless, additional studies are required to evaluate the cross-subtype recognition of the selected Gag and Nef antibodies across diverse HIV-1 subtypes.

Please refer to Page 10 and lines 196-203 in the revised manuscript for the details.

6. *Typo in S1 Figure A: panel 1 should read “at RT”.*

- **Response:** The suggested change has been made to the figure.

7. *Possible typo S1 Figure A: panel 2 says “1 ~2M cells per well”. Should this be “1-2M cells per well” or “~2M cells per well”.*

- **Response:** The suggested change has been made to the figure.

8. *Typo in S1 Figure B: step 1.4 should read “…1,500 rpms…”.*

- **Response:** The suggested change has been made to the figure.

9. *S1 figure B: step 1.6: consider amending the text to “prepare 2 million cells in 3mL GM per coverslip”.*

- **Response:** The original text in S1 Figure B has revised.

10. *Given that the two anti-Nef antibodies cannot be used simultaneously to stain the same slide, what are the authors recommendations for modifying the assay to be used with clinical samples so that intact- and defective- genome-carrying cells can be differentiated on the same slide? I realize this may not be the primary purpose of this assay, but it would be poignant to mention how this assay could be of benefit in assessing the reservoir in clinical samples (or even discuss whether it would be useful in the context of deletions in the proviral population since all defective proviruses are not the result of point mutations).*

- **Response:** We are grateful for this constructive comment. And agree that using all antibodies on the same sample would be ideal. However, both anti-Nef antibodies (3D12 and EH1) are mouse IgG1, so with the currently available reagents it is not possible to distinguish them using standard secondary antibodies. In the future, if clones from different species or IgG isotypes become available, the assay could be adapted to allow simultaneous detection of Nef and Gag on the same slide, which would be more convenient for clinical applications.

Although the current assay has certain limitations, it still provides valuable insights into the composition of the viral reservoir in clinical samples: cells expressing Nef (3D12) are likely enriched for intact proviruses, whereas cells lacking Nef (detected by 3D12 or EH1) may represent defective proviruses. These considerations highlight the potentials of this assay in future studies aimed at assessing the HIV reservoir in patients.

We have included the discussion in the final paragraph of the revised manuscript to highlight the rational of the established assay. please see Pages 10-11 and lines 204-216 in the revised manuscript for the updates.
