# Supplementary material for: An immunofluorescence microscopy assay to discriminate distinct expression patterns of HIV-1 Gag and Nef proteins in HIV-1 provirus-harboring cells
Source: PLoS One. 2026 Jan 16;21(1):e0340463. doi: 10.1371/journal.pone.0340463 (PMC12810823; doi:10.1371/journal.pone.0340463)
Supplement: Attachment — Submitted filename: PONE-D-25-53504 reviewer comments.docx [file pone.0340463.s003.docx]

Reviewer comments:

Overview: This is a timely and interesting methods paper given the emerging picture of the clinical relevance of defective proviruses in the HIV-1 latent reservoir. The paper describes a staining protocol using two antibodies to HIV-1 Nef that can differentiate proteins produced from intact and defective HIV-1 proviruses in combination with an anti-HIV-1 Gag antibody. The manuscript is concise but clear and was a pleasure to read. The protocol is clear and easy to understand with a good level of detail for replicating the experiments in other settings. Most of the points in the revision document are recommend small changes (like typos) to the manuscript and supplementary information. However, point 10 is important to note for translation of this protocol into one that can be used with clinical samples.

1. Line 39 (optional): Suggestion to change the word “targeted” to “removed” or a synonym. There is some (*ex vivo*) evidence of CTL recognition of cells with defective genomes if proteins are expressed, even if that targeting does not effectively clear the reservoir in people with HIV (DOI: [10.1016/j.chom.2017.03.008](https://doi.org/10.1016/j.chom.2017.03.008) ).
2. Line 67: Please add the catalogue number for H9NM cells (ARP-402).
3. Line 76: Please change “reach to “reached”.
4. Line 82: Kindly remember to include the Protocols.io link in the final version.
5. Can the authors please add a caveat to the conclusion about cross-subtype recognition of the Gag and Nef antibodies used in this experiment? This speaks directly to the how broadly this assay can be used to characterize cells harbouring intact and defective proviruses across HIV subtypes which are distributed geographically.
6. Typo in S1 Figure A: panel 1 should read “at RT”.
7. Possible typo S1 Figure A: panel 2 says “1 ~2M cells per well”. Should this be “1-2M cells per well” or “~2M cells per well”.
8. Typo in S1 Figure B: step 1.4 should read “…1,500 rpms…”.
9. S1 figure B: step 1.6: consider amending the text to “prepare 2 million cells in 3mL GM per coverslip”.
10. Given that the two anti-Nef antibodies cannot be used simultaneously to stain the same slide, what are the authors recommendations for modifying the assay to be used with clinical samples so that intact- and defective- genome-carrying cells can be differentiated on the same slide? I realize this may not be the primary purpose of this assay, but it would be poignant to mention how this assay could be of benefit in assessing the reservoir in clinical samples (or even discuss whether it would be useful in the context of deletions in the proviral population since all defective proviruses are not the result of point mutations).
